# Undiagnosed Celiac Disease Associated With Antiphospholipid Syndrome Causing Infertility and Osteoporosis

**DOI:** 10.7759/cureus.49899

**Published:** 2023-12-04

**Authors:** Aljoharah Al Saud, Ziad F Rayes

**Affiliations:** 1 Family Medicine and Polyclinics, Alfaisal University College of Medicine, Riyadh, SAU; 2 Family Medicine and Polyclinics, King Faisal Specialist Hospital and Research Centre, Riyadh, SAU

**Keywords:** autoimmune disease, osteoporosis, infertility, antiphospholipid syndrome, celiac disease

## Abstract

Celiac disease and antiphospholipid syndrome are two conditions associated with infertility, but their coexistence is rarely reported. In this manuscript, we present the case of a 23-year-old woman initially presenting with urticaria and vitamin D deficiency, subsequently developing recurrent miscarriages and osteoporosis over a period of 13 years. After initially presenting with urticaria and vitamin D deficiency, she was diagnosed with idiopathic urticaria. Thirteen years later, the patient was diagnosed with antiphospholipid syndrome following multiple pregnancy losses and eventually underwent in vitro fertilization successfully with a diamniotic dichorionic pregnancy. Post-delivery, the patient developed severe back pain, due to underlying acute wedge fractures indicative of osteoporosis. Further investigations revealed elevated antigliadin and anti-tissue transglutaminase antibodies, leading to a diagnosis of celiac disease. She responded well to a gluten-free diet with significant symptomatic and bone mass density improvements. This case highlights the importance of considering celiac disease in cases of unexplained infertility and osteoporosis. Moreover, it emphasizes the need for early diagnosis of celiac disease to minimize its detrimental effects on fertility and bone health.

## Introduction

Celiac disease is among the most common autoimmune gastrointestinal disorders worldwide. A meta-analysis found that the seropositive-celiac disease is prevalent in 15.6% of high-risk individuals in Saudi Arabia [1]. Celiac disease commonly manifests with diarrhea, loss of appetite, bloating, and iron deficiency anemia in both children and adults [2]. Undiagnosed celiac disease is a risk factor for infertility in woman [3]. However, celiac disease is frequently overlooked as a differential diagnosis in the management of infertility.

Antiphospholipid syndrome is a common cause of infertility characterized by thrombotic events and pregnancy morbidity. Although antiphospholipid syndrome often presents as a primary entity, cases reporting its coexistence with celiac disease are rare [4,5].

In addition, some have hypothesized that antiphospholipid syndrome is one of the pathogenic mechanisms of celiac disease leading to infertility [6]. Hence, we report the case of a woman with chronic undiagnosed celiac disease and coexisting controlled-antiphospholipid syndrome leading to infertility and osteoporosis. We aim to highlight the importance of considering celiac disease as a differential in the management of infertility among women.

## Case presentation

A 23-year-old female presented to the immunology clinic for a four-month history of progressive urticaria. Labs revealed a mildly elevated erythrocyte sedimentation rate and vitamin D deficiency. She was diagnosed with idiopathic urticaria and prescribed cetirizine 10 mg at bedtime and loratadine during the day as needed with major improvements. The patient was also started on vitamin D two tablets daily of 800 IU and one tablet of calcium 600 mg for three months.

Thirteen years later, the patient was admitted to the obstetrics ward at 11 weeks of gestation due to failed medical management of a missed miscarriage. Bedside ultrasound revealed a small intrauterine gestational sac with no fetal ball. The patient received several courses of misoprostol with no success and, therefore, underwent dilatation and curettage. The patient was discharged the next day with no complications. Four months later, she presented once again at seven weeks of gestation with vaginal bleeding, and an ultrasound revealed an empty uterus, confirming a complete abortion.

An autoantibody panel revealed elevated levels of anti-cardiolipin IgM of 123.3 MPL, anti-B2-glycoprotein I IgM of 53.6 SMU, anti-phosphatidylserine IgM >100 MPS U/mL, and rheumatoid factor IgM of 99.6 units. Chromosomal analysis was normal in the couple. Coagulation studies revealed elevated prothrombin time of 14.6 seconds, partial thromboplastin time of 41.1 seconds, and protein C levels of 1.3 IU/mL and reduced functional protein S levels of 0.28 IU/mL. The patient was accordingly diagnosed with antiphospholipid syndrome.

Three months later, the patient successfully underwent in vitro fertilization with twin dichorionic diamniotic gestation. She was prescribed aspirin 81 mg daily, ferrous gluconate 324 mg, heparin 7500 units twice daily, and hydroxychloroquine twice daily. However, the patient presented at 24 weeks of gestation with fatigue, headache, dizziness, and dyspnea for three days. Blood labs revealed a hemoglobin level of 75 g/L, hematocrit of 0.223, mean corpuscular volume (MCV) of 85.8 fL, mean corpuscular hemoglobin (MCH) of 28.8 pg, and a corrected reticulocyte count of 0.6%. Further tests revealed a ferritin level of 31 ug/L and a decreased vitamin B12 level of 144 pmol/L. The patient was diagnosed with hematinic deficiency and given hydroxocobalamin 1 mg and ferric carboxymaltose 750 mg. The patient successfully underwent a C-section at 34 weeks with no complications, and both neonates are currently alive and well.

Approximately a month after delivery, the patient began reporting severe back pain in the lower back. Her biochemical profile was completely normal including parathyroid hormone, vitamin D, thyroid-stimulating hormone (TSH), bone profile, and inflammatory markers. The patient was diagnosed as a case of pregnancy-induced osteoporosis and given teriparatide 20 mg subcutaneously daily.

**Figure 1:**
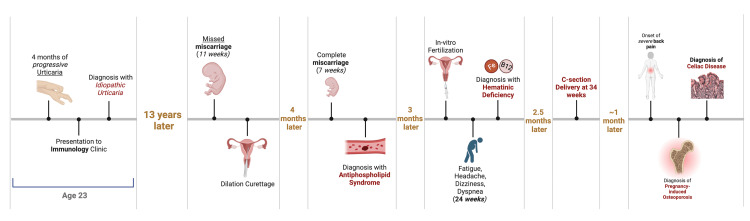
Graphic representation of the patient's chronological timeline from the beginning of the case to the end BioRender.com was used to create the figure.

Although the patient reported moderate improvement in symptoms, symptoms persisted in the thoracic region. The patient was then suspected of celiac disease, and an antibody panel revealed elevated anti-gliadin IgG levels of 31.4 mg/L, anti-gliadin IgA levels of 12.4 mg/L, and anti-tissue transglutaminase IgA levels of 191.2 units. The patient subsequently underwent an endoscopic procedure complemented by a biopsy, which confirmed the diagnosis of celiac disease. The patient is currently following a gluten-free diet with symptomatic and bone mineral density (BMD) improvement. A graphic representation of the patient's timeline is visualized in Figure 1.

## Discussion

In this report, we present a complex case of a young woman with undiagnosed celiac disease associated with antiphospholipid syndrome, leading to infertility and osteoporosis. This case underscores the importance of considering celiac disease as a potential underlying condition in cases of unexplained infertility and osteoporosis, despite its frequent omission from the list of differentials.

Celiac disease and antiphospholipid syndrome can individually contribute to infertility [3,7,8]. However, their coexistence, as demonstrated in this case, can lead to an enhanced risk profile. Although the association between celiac disease and antiphospholipid syndrome is rare, this case suggests that when they coexist, they can synergistically affect fertility. Jorge et al. reported three cases of women diagnosed with celiac disease presenting with a spectrum of fetal death, transient ischemic attacks, and thrombosis [6]. Notably, however, the patients were diagnosed with celiac disease prior to their diagnosis of antiphospholipid syndrome [6]. On the contrary, our patient was diagnosed with antiphospholipid syndrome due to recurrent abortions, after which a compression fracture led to the diagnosis of celiac disease. A study by Karoui et al. found that celiac disease patients had significantly higher levels of anticardiolipin IgA levels compared to control [9]. However, only two (4%) patients suffered thrombotic and pregnancy complications, and neither events were related to antiphospholipid syndrome-associated antibodies [9]. The hypothesized pathogenic mechanism of antiphospholipid syndrome being a driving factor of infertility in celiac disease, as proposed by previous studies [6], warrants further exploration to further understand this association. However, it is important to note that certain studies in the literature suggest that the prevalence of celiac disease in infertile women is not higher than in the general population [8].

Another striking feature of this case is the patient's missed and late diagnosis of celiac disease, despite exhibiting signs of possible celiac disease early at presentation (urticaria and vitamin D deficiency). The late diagnosis may have amplified the negative effects of both celiac disease and antiphospholipid syndrome on fertility and bone health, exacerbating the severity of the patient's condition. Therefore, it is crucial to consider celiac disease among the differential diagnoses of infertility, especially in patients with chronic nutritional deficiencies.

## Conclusions

This case brings to light the multifaceted role of celiac disease and the significance of early diagnosis. It underscores the need for healthcare providers to maintain a high degree of suspicion for celiac disease in patients presenting with unexplained infertility or osteoporosis, particularly if other signs of autoimmune diseases and vitamin deficiencies are present. Given the potential associations between celiac disease, antiphospholipid syndrome, and infertility, further research is required to elucidate these complex relationships and to refine guidelines for screening and management.
